# MIL-101 (Fe)-NH_2_ metal-organic framework/graphene oxide nanocomposite modified screen-printed carbon electrode for electrochemical sensing of 2,4-dichlorophenol in water samples

**DOI:** 10.1016/j.heliyon.2025.e42285

**Published:** 2025-01-25

**Authors:** Hadi Beitollahi, Somayeh Tajik, Fariba Garkani Nejad

**Affiliations:** a Environment Department, Institute of Science and High Technology and Environmental Sciences, Graduate University of Advanced Technology, Kerman, Iran; b Research Center of Tropical and Infectious Diseases, Kerman University of Medical Sciences, Kerman, Iran

**Keywords:** 2,4-dichlorophrnol, Screen-printed carbon electrode, Electrochemical sensing platform, Graphene oxide, MIL-101 (Fe)-NH_2_ metal-organic framework

## Abstract

In the present study, we report the synthesis of MIL-101 (Fe)-NH_2_ metal-organic framework/graphene oxide (MIL-101 (Fe)-NH_2_ MOF/GO) nanocomposite, which was synthesized through simple solvothermal method. Various characterization techniques including Field-emission scanning electron microscopy (FE-SEM), Energy dispersive X-ray spectroscopy (EDX), and X-ray diffraction (XRD) were employed for the morphological and structural characterization of the synthesized nanocomposite. Then, the prepared MIL-101 (Fe)-NH_2_/GO nanocomposite was drop-casted on the screen-printed carbon electrode (SPCE) to fabricate a simple, rapid, and sensitive electrochemical sensing platform for *2*,*4*-*dichlorophenol (2,4-DCP)* determination in the water samples. Comparative analysis using cyclic voltammetry (CV) showed that the MIL-101 (Fe)-NH_2_/GO/SPCE significantly improved the oxidation reaction of 2,4-DCP with observation of higher detection current at lower over-potential compared to unmodified SPCE. This observation can be related to the synergistic combination of MIL-101 (Fe)-NH_2_ MOF and GO sheets. The linear response of MIL-101 (Fe)-NH_2_/GO/SPCE sensor for determining 2,4-DCP using voltammetric measurements was observed in the range of 0.001–440.0 μM with a low detection limit (LOD) of 0.5 nM and a high sensitivity of 0.2026 μA μM^−1^. Finally, the modified SPCE efficiently exhibits its high accuracy in detecting 2,4-DCP in water samples, demonstrating remarkable recovery percentages of 97.5–104.4 %.

## Graphical abstract

A novel electrochemical sensor for determination of 2,4-dichlorophenol in water samples was constructed.

**Figure:**
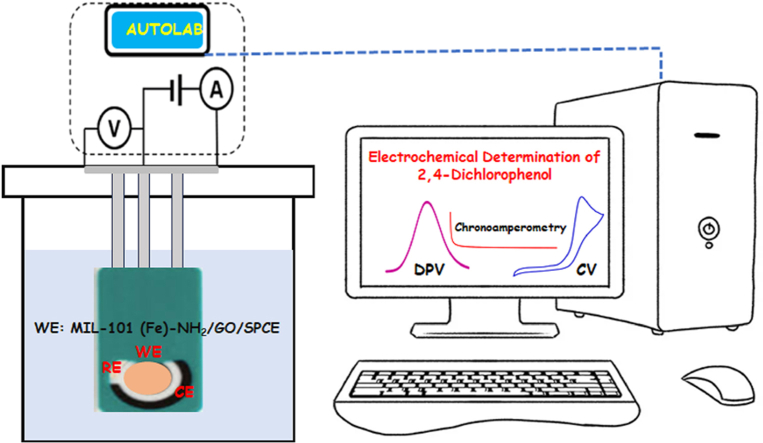

## 1 Introduction

Nowadays, the environmental pollution is considered as one of the most important issues. The level of environmental pollutants in today's industrial world is constantly increasing with the progress of technologies and population growth [1]. Pollutants can enter the food chain through soil, water or agricultural systems and endanger food safety and human health. Therefore, the control of environmental pollution is very important. The recent studies have demonstrated that phenol and its derivatives are classified as major pollutants present in the environment [2]. 2,4-dichlorophenol (2,4-DCP) is one of the chlorinated phenols which is utilized in the production of pharmaceuticals, herbicides, insecticides, fungicides, disinfectants, dyes, plastics, and etc. [3,4]. With the wide application of 2,4-DCP in medicine, industry, and agriculture, it is discharged into water bodies, resulting in significant damage to the both environment and human health due to its high toxicity, low biodegradability, and high accumulation in the environment [5]. 2,4-DCP can cause serious and various health problems, including probable mutagenic and carcinogenic effects, respiratory infections, and damage to kidneys, liver, skin, lungs, and etc. [[6], [7], [8]]. Therefore, the first and important step in controlling the level of environmental pollution through 2,4-DCP is the detection of this compound. For this purpose, advanced tools are needed that can detect even very low amounts of this compound in the water samples. So far, various methods including gas chromatography (GC) [9], high-performance liquid chromatography (HPLC) [10], chemiluminescence [11], colorimetry [12], capillary electrophoresis [13], and electrochemistry [[14], [15], [16], [17]] have been extensively reported to detect 2,4-DCP in the water samples. However, complex and high-cost equipment, very long analysis time, skilled operators, tedious and complicated experimental procedures (such as pre-treatment processes), and etc. are usually needed to perform some of these methods. Among these methods, analysis based on electrochemical methods provided simple, fast, and low-cost detections along with high sensitivity and selectivity in various areas including environmental monitoring, food safety, and analysis of biomarkers and pharmaceutical compounds [[18], [19], [20], [21], [22], [23]]. In addition, electrochemical methods have the capability to perform on-site analyses.

Screen-printed electrodes (SPEs) are a type of printed electrodes that are produced through the utilization of screen-printing technique. The recent electrochemical studies revealed that SPEs emerged as reliable, economical, portable, and practical platforms in electrochemical sensing applications [[24], [25], [26], [27]]. It should be noted that the application of unmodified electrodes in electro-analysis of compounds exhibits limitations such as high over-potential, slow electron transfer, limited catalytic activity, and insufficient selectivity and sensitivity for detections. Utilizing advanced technologies including molecular engineering, nanotechnology, and etc. can provide new solutions to overcome the limitations for the application of unmodified electrodes.

Nanotechnology, as one of the emerging and advancing technologies, has witnessed significant advancements in diverse areas including medicine, materials science, electronics, energy, and environmental protection [[28], [29], [30], [31], [32], [33], [34], [35], [36], [37], [38], [39]]. In particular, the recent studies have shown that nanotechnology can serve as a powerful tool in improving the performance of electrochemical sensors [[40], [41], [42], [43], [44]]. This technology enables researchers to design electrochemical sensors with prominent features such as large surface area, high porosity, and desirable electrical conductivity [[45], [46], [47], [48]].

As a new class of highly porous crystalline materials composed of metal ions and organic linkers, metal-organic frameworks (MOFs), also known as porous coordination polymers (PCPs), have attracted broad attention from researchers in the recent decades. The amalgamation of metal ions and organic linkers in MOFs provides the opportunity to engineer and design materials that possess customized properties and functionalities. Owing to the unique features (including large surface areas, rich and accessible active sites, high porosity structure, tailorable pore sizes, structural diversity, and easy functionalization process), these frameworks exhibit functional ability and applicability in diverse fields (including biomedicine, catalysis, gas separation, energy storage and conversion, and sensing applications) [[49], [50], [51], [52], [53], [54], [55]]. In order to enhance the characteristics of MOFs, such as conductivity, stability, and catalytic performance, MOFs can be effectively combined or supported with various functional materials, including carbon nanostructures, metal and metal oxide nanoparticles, conductive polymers, and etc. [[56], [57], [58]]. These composite structures of MOFs provide unique properties that are unattainable in pure MOFs. Graphene oxide (GO), the oxidized derivative of graphene, is a two-dimensional (2D) carbon nanostructure with a hexagonal lattice structure that consists of a single layer of carbon atoms and oxygen-containing functional groups on its surface. Due to the large surface area, good electrical conductivity, oxygen-containing functional groups, chemical stability, and tunable properties, GO demonstrated applications in various electrochemical fields. This carbon nanostructure has been effectively utilized in the design and fabrication of supercapacitors, electrochemical sensors and biosensors, and other electrochemical devices [[59], [60], [61], [62]]. The unique properties of GO enable researchers to enhance the electrochemical performances and contribute to the development of advanced technologies in energy storage, sensing and catalysis.

Herein, a simple and sensitive electrochemical sensing platform based on nanocomposite of MIL-101 (Fe)-NH_2_ MOF and GO has been developed for improved determination of 2,4-DCP in the water samples. The MIL-101 (Fe)-NH_2_/GO/SPCE sensing platform demonstrated better electrochemical performance than unmodified SPCE towards 2,4-DCP determination due to its higher electrical conductivity and larger active surface area. Based on quantitative measurements, the developed sensor demonstrated favorable electrochemical sensing performance for 2,4-DCP determination, including high sensitivity in a wide linear detection range with low LOD. In addition, the analysis of water samples revealed the efficient performance of the designed sensor in this work. The major novelty of the developed sensor in the present work lies in the utilization of MIL-101 (Fe)-NH_2_/GO nanocomposite with good properties as an efficient composite for modification of SPCE towards voltammetric determination of 2,4-DCP. Based on the conducted research, the developed sensor can be used as a valuable tool in the environmental monitoring and ensuring water safety.

## 2 Experimental

### 2.1 Materials and instruments

The analytical grade of chemicals was provided with highest level of purity from companies such as Merck and Sigma-Aldrich, and were utilized without extra purifications.

The electrochemical techniques including chronoamperometry and voltammetry (differential pulse voltammetry (DPV) and CV) were conducted by using Autolab Potentiostat – Galvanostat (PGSTAT 302N-Metrohm (Netherlands). The SPCEs (DRP-110 model-Dropsens (Spain)) were used in all electrochemical experiments. Each SPCE consists of a three-electrode system (carbon working electrode, carbon counter electrode, and Ag pseudo reference electrode). The supporting electrolyte (phosphate buffer solution (PBS) – 0.1 M) with different values of pH were prepared using pH meter (type 713-Metrohm (Switzerland)). The aqueous solutions of reagents and buffer solutions were prepared using Water Purification System (Direct-Q® 8 UV-Millipore (Gemany)).

Field-emission scanning electron microscope (MIRA3-TESCAN (Czech Republic)) with EDX detector instrument, and X-ray diffractometer (X'PERT PRO-Panalytical (Netherlands)) were applied to study the morphological and structural characterizations of MIL-101 (Fe)-NH_2_ MOF/GO nanocomposite.

### 2.2 Synthesis of MIL-101 (Fe)-NH_2_ MOF/GO nanocomposite

Based on the previous report by Su et al. with some changes, the solvothermal synthesis of MIL-101 (Fe)-NH_2_ MOF/GO nanocomposite was carried [63]. For this purpose, 1.24 mmol of 2-aminoterephthalic acid (0.224 g) and 2.48 mmol of FeCl_3_.6H_2_O (0.670 g) were dissolved into 20 mL N,N-dimethylformamide (DMF) under magnetic stirring for 10 min at ambient temperature. Then, 0.006 g of GO was dispersed into the above solution and ultrasonicated for 40 min. After ultrasonication, this suspension was transferred into a Teflon (polytetrafluoroethylene)-lined stainless-steel autoclave and subjected to a solvothermal reaction at 110 °C for 24 h into an oven. After that, the autoclave was taken out of the oven and was cooled to room temperature. After cooling, the prepared precipitate was collected through centrifugation and washed with ethanol and DMF for several times. Finally, the product was dried in a vacuum oven at 65 °C for 15 h.

### 2.3 Modification of SPCE

The MIL-101 (Fe)-NH_2_/GO nanocomposite modified SPCE was utilized for electrochemical investigations. The modification of SPCE was performed based on the previous studies [[64], [65], [66]]. Therefore, for modification of SPCE, 1 mg/mL of MIL-101 (Fe)-NH_2_/GO suspension was firstly prepared in deionized water by ultrasonication for 20 min. Then, 3.0 μL of ultrasonicated suspension of MIL-101 (Fe)-NH_2_/GO was drop-casted on SPCE surface. After that, it was dried (room temperature for 20 min).

## 3 Results and discussion

### 3.1 Characterization of MIL-101 (Fe)-NH_2_ MOF/GO nanocomposite

**Fig. 1:**
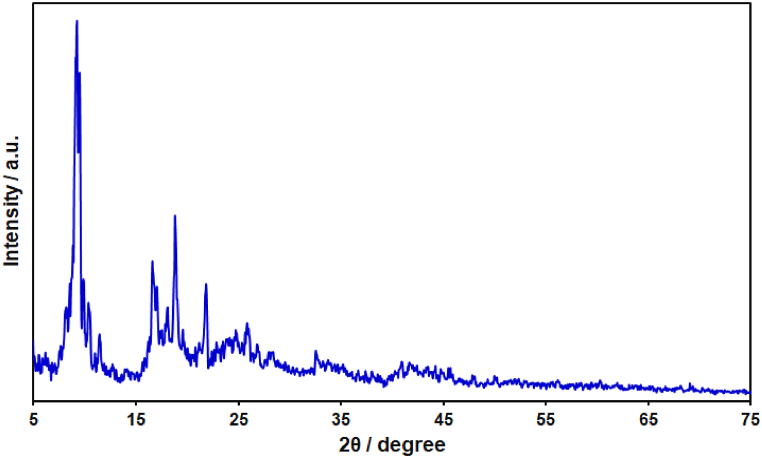
XRD pattern of MIL-101 (Fe)-NH_2_ MOF/GO nanocomposite.

In order to characterize the crystalline structure of the MIL-101 (Fe)-NH_2_ MOF/GO nanocomposite, the XRD characterization was used. Fig. 1 shows the XRD pattern of nanocomposite. The observed diffraction peaks in the XRD pattern are related to the MIL-101 (Fe)-NH_2_ MOF. The observed diffraction peaks correspond to the previously reported XRD patterns of MIL-101 MOF (CCDC 605510) [67,68]. In addition, the diffraction peaks of GO probably overlapped or covered with the characteristic peaks of prepared MOF.

**Fig. 2:**
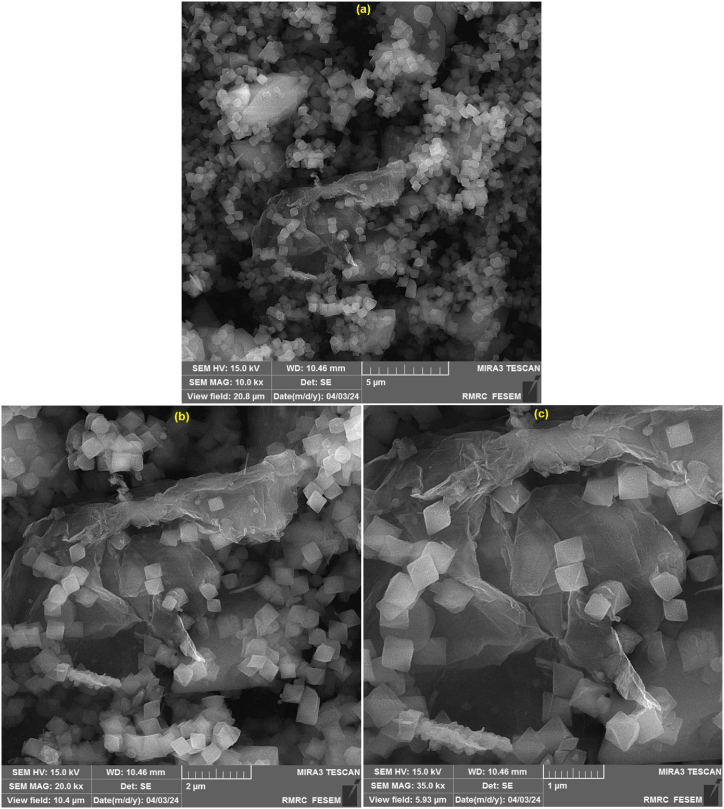
FE-SEM images of MIL-101 (Fe)-NH_2_ MOF/GO nanocomposite at a (5 μm), b (2 μm), and c (1 μm) magnifications.

Then, the FE-SEM images were obtained to characterize the structure and morphological characteristics of MIL-101 (Fe)-NH_2_ MOF/GO nanocomposite. The FE-SEM images of the prepared nanocomposite at 5 μm, 2 μm, and 1 μm magnifications are presented in Fig. 2a–c respectively. As can be seen, the FE-SEM images reveal the formation of MIL-101 (Fe)-NH_2_ MOF with octahedral morphology on the surface of GO sheets. The FE-SEM images clearly show the existence of both MIL-101 (Fe)-NH_2_ MOF and GO in the prepared nanocomposite. Also, the observed octahedral morphology for MIL-101 (Fe)-NH_2_ MOF is in good agreement with previous reports [63,69,70].

**Fig. 3:**
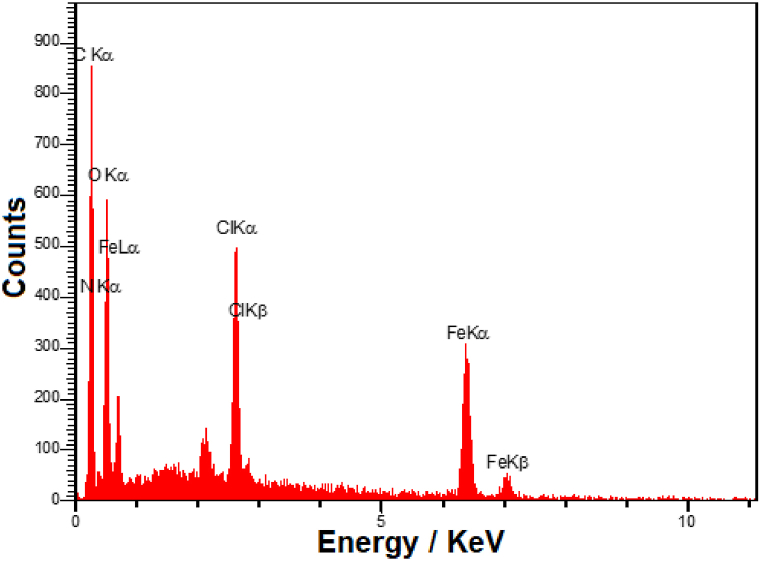
EDX spectrum of MIL-101 (Fe)-NH_2_ MOF/GO nanocomposite.

Furthermore, the EDX analysis was performed to investigate the elemental composition of the as-synthesized nanocomposite. It can be seen from the EDX spectrum (Fig. 3), the characteristic peaks of C, Fe, O, N, and Cl elements were detected in the composition of MIL-101 (Fe)-NH_2_ MOF/GO nanocomposite. According to previous reports, the MIL-101 (Fe)-NH_2_ MOF contains Fe, C, O, N, and Cl elements [71,72].

### 3.2 Electrochemical investigation of 2,4-DCP at the unmodified SPCE and MIL-101 (Fe)-NH_2_/GO/SPCE

The pH value of supporting electrolyte has a significant influence on the performance of sensors in the electroanalysis of various compounds. Therefore, the effect of pH on the response of MIL-101 (Fe)-NH_2_/GO/SPCE towards 50.0 μM 2,4-DCP determination was estimated with changing the pH value of 0.1 M PBS between 3.0 and 9.0. The obtained results from DPV demonstrated that changing pH of PBS significantly affected the response of the modified SPCE. There was an increase in current response when pH value increased from 3.0 to 7.0. In fact, a significant current response of MIL-101 (Fe)-NH_2_/GO/SPCE was observed at pH = 7.0 of PBS, showing that the electrochemical oxidation of 2,4-DCP is very favorable. It should be noted that by increasing the pH value (at pH values higher than 7.0), the current response began to decrease. Hence, pH = 7.0 of PBS (0.1 M) was chosen as the best conditions of pH.

**Fig. 4:**
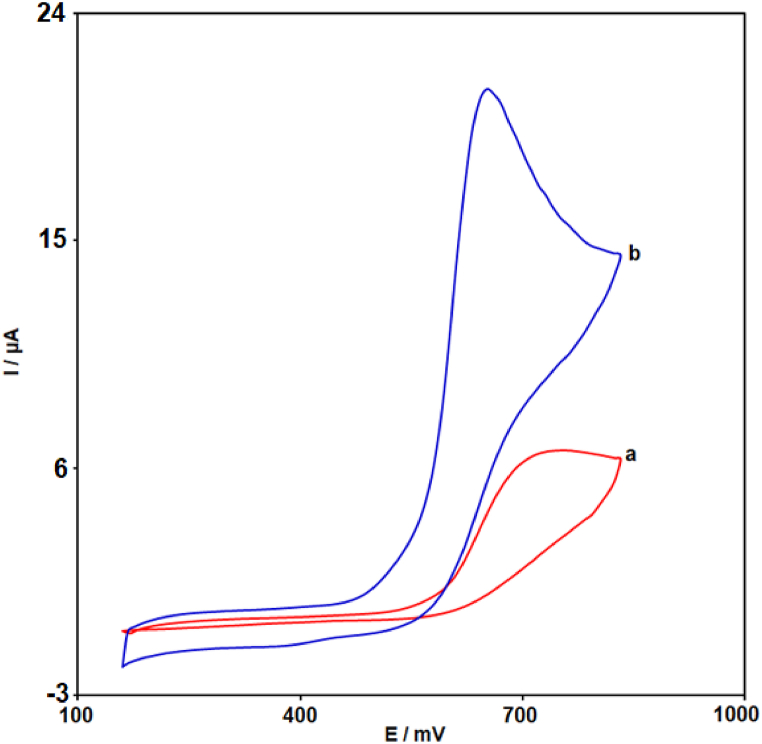
CV responses of unmodified SPCE (cyclic voltammogram-a) and MIL-101 (Fe)-NH_2_/GO/SPCE (cyclic voltammogram-b) in PBS (0.1 M with pH 7.0) containing 2,4-DCP (100.0 μM) at 50 mV/s (scan rate).

To evaluate the response of unmodified SPCE and MIL-101 (Fe)-NH_2_ MOF/GO modified SPCE towards 100.0 μM 2,4-DCP in PBS (0.1 M with pH 7.0), CV was applied (Fig. 4). Comparing the recorded cyclic voltammograms demonstrated that the unmodified SPCE has a lower current response (anodic peak current (Ipa) = 6.8 μA) to 2,4-DCP at higher overpotential (anodic peak potential (Epa) = 745 mV) than modified SPCE. However, the oxidation overpotential of 2,4-DCP at MIL-101 (Fe)-NH_2_/GO/SPCE decreased significantly (Epa = 650 mV), and the current response enhanced obviously (Ipa = 21 μA). The significant reduction of Epa along with enhancement of Ipa of 2,4-DCP can be related to the synergistic effects of GO and MIL-101 (Fe)-NH_2_ MOF.

### 3.3 Investigation of scan rate

**Fig. 5:**
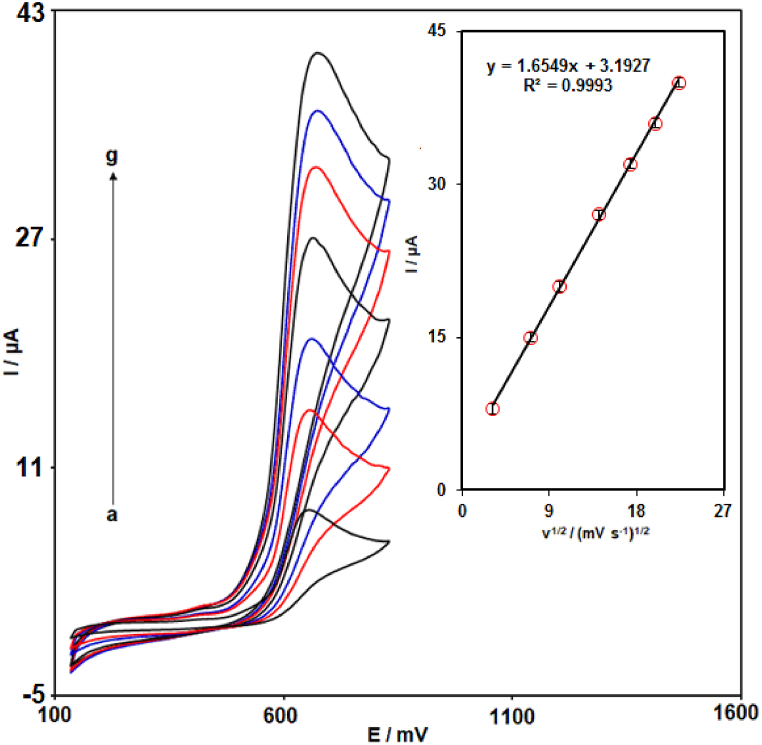
CV responses of MIL-101 (Fe)-NH_2_/GO/SPCE in 0.1 M PBS with pH 7.0 containing 70.0 μM 2,4-DCP at scan rates of (a) 10, (b) 50, (c) 100, (d) 200, (e) 300, (f) 400, and (g) 500 mV/s. Inset: Corresponding linear plot of Ipa (μA) vs. υ^1/2^ (mV/s)^1/2^.

CV studies were also carried out to evaluate the electrochemical response of MIL-101 (Fe)-NH_2_/GO/SPCE towards 70.0 μM 2,4-DCP in PBS (0.1 M with pH = 7.0) at various scan rates. The recorded cyclic voltammograms are illustrated in Fig. 5. As shown by the recorded cyclic voltammograms, the Ipa of 2,4-DCP improved with scan rate increasing. Also, as seen in Inset of Fig. 5, a good linear relationship was observed between Ipa of 2,4-DCP and the square root of scan rate with the regression equation as follow: Ipa (μA) = 1.6549υ^1/2^ (mV s^−1^)^1/2^ + 3.1927 (R^2^ = 0.9993). It was demonstrated that the oxidation of 2,4-DCP at MIL-101 (Fe)-NH_2_/GO/SPCE was a diffusion-controlled process.

### 3.4 Chronoamperometric study of 2,4-DCP at MIL-101 (Fe)-NH_2_/GO/SPCE

**Fig. 6:**
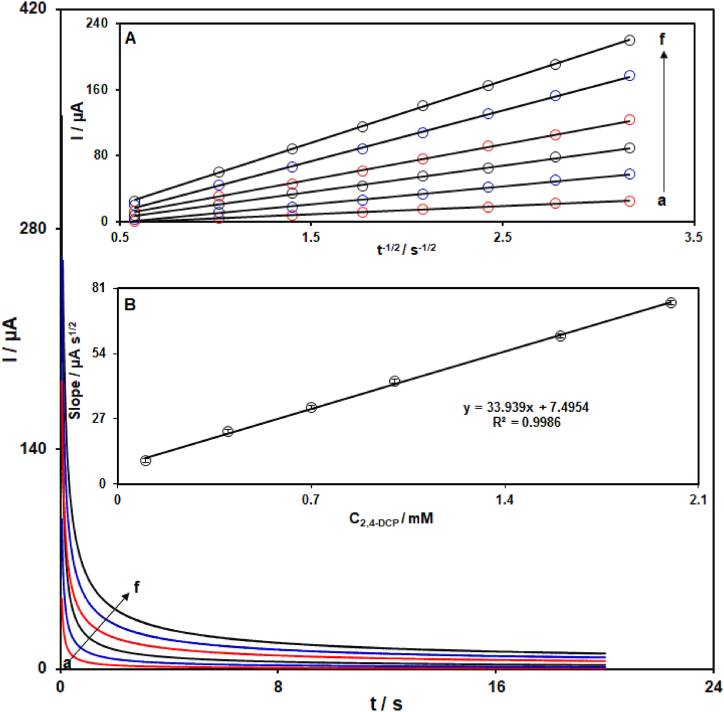
Chronoamperometric responses of MIL-101 (Fe)-NH_2_/GO/SPCE in PBS (0.1 M with pH 7.0) with various concentrations of 2,4-DCP (chronoamperograms a to f related to 0.1, 0.4, 0.7, 1.0, 1.6, and 2.0 mM). Insets: Linear dependence of current versus t^−1/2^ from chronoamperometric measurements (A) and Linear dependence of slopes from I-t^−1/2^ plots versus concentrations of 2,4-DCP (B).

The chronoamperometry study has been performed to determine the diffusion coefficient (D) of 2,4-DCP at MIL-101 (Fe)-NH_2_/GO/SPCE. Fig. 6 illustrates the chronoamperometric responses of modified SPCE in PBS (0.1 M with pH 7.0) containing 2,4-DCP at various concentrations with a step-potential of 700 mV. The resulting chronoamperograms demonstrate the dependence of current on time (t). From the chronoamperograms, with the progress of time, the current has decreased. The D for 2,4-DCP can be determined through the Cottrell equation of I = nFACD^1/2^/π^1.2^t^1/2^ [73], where D represents the diffusion coefficient (cm^2^/s), I represents the current response (μA), F represents the Faraday's constant (96,485C mol^−1^), A represents the surface area of electrode (cm^2^), n represents the number of transferred electrons in the oxidation reaction of 2,4-DCP, C represents the concentration of 2,4-DCP (mol/cm^3^), and t represents the time (s). The experimental plots for linear variations of Ipa against t^−1/2^ were shown in Fig. 6-Inset A. Then, a straight line was obtained via plotting the resulting slopes from I-t^−1/2^ plots against concentrations of 2,4-DCP)(Fig. 6-Inset B) . In accordance with Cottrell equation and the resulting slope from Slope-C_2,4-DCP_ plot, the D of 2,4-DCP was estimated at about 9.8 × 10^−5^ cm^2^/s.

### 3.5 Quantitative determination of 2,4-DCP at MIL-101 (Fe)-NH_2_/GO/SPCE by DPV

**Fig. 7:**
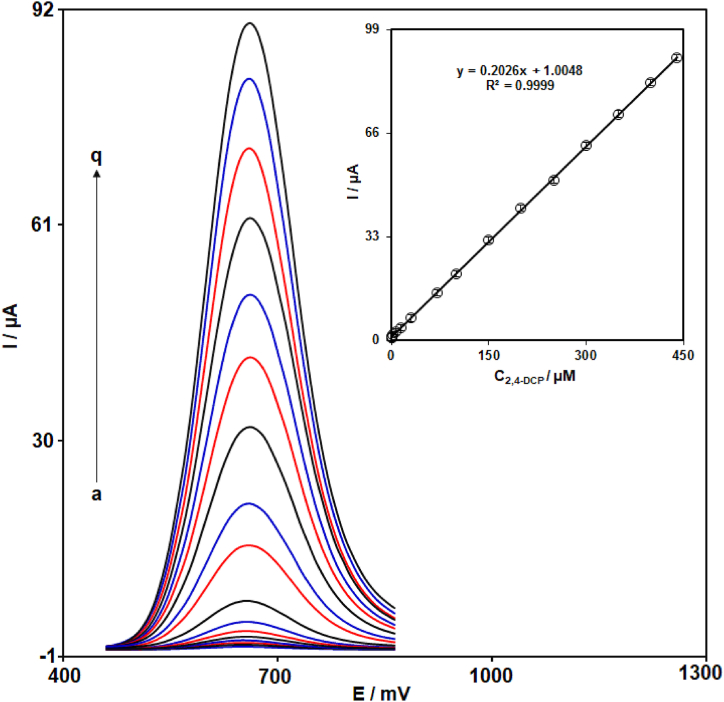
DPV responses of MIL-101 (Fe)-NH_2_/GO/SPCE in PBS (0.1 M with pH 7.0) containing 2,4-DCP at concentrations of (a) 0.001, (b) 0.01, (c) 0.1, (d) 1.0, (e) 2.5, (f) 7.5, (g) 15.0, (h) 30.0, (i) 70.0, (j) 100.0, (k) 150.0, (l) 200.0, (m) 250.0, (n) 300.0, (o), 350.0, (p) 400.0, and (q) 440.0 μM. Inset: Corresponding calibration plot of Ipa (μA) vs. C_2,4-DCP_ (μM).

For determination of various concentrations of 2,4-DCP at MIL-101 (Fe)-NH_2_/GO/SPCE, DPV method was used for quantification of 2,4-DCP. Fig. 7 illustrates the DPV responses of MIL-101 (Fe)-NH_2_/GO/SPCE for 2,4-DCP at different concentrations (0.001 μM–440.0 μM) in PBS (0.1 M with pH = 7.0). As showed in Fig. 7, the current responses of modified SPCE gradually enhanced with the increase in the 2,4-DCP concentrations. Also, a linear relationship between the current responses of MIL-101 (Fe)-NH_2_/GO/SPCE and 2,4-DCP concentrations in the range of 0.001 μM–440.0 μM was obtained (regression equation = Ipa (μA) = 0.2026C_2,4-DCP_ (μM) + 1.0048 (R^2^ = 0.9999)) (Inset of Fig. 7). The LOD was determined through the equation of LOD = 3 S/m, where S indicates the standard deviation (SD) of current responses of developed sensor for blank solution (PBS 0.1 M-pH 7.0) without the presence of 2,4-DCP and m indicated the slope of calibration plot. Based on the above equation, the LOD was determined to be 0.5 nM. Also, a comparison of developed sensor (MIL-101 (Fe)-NH_2_/GO/SPCE) with the previously reported electrochemical sensors is given in Table 1. The comparative investigation in Table 1 shows the good analytical performance of MIL-101 (Fe)-NH_2_/GO/SPCE for voltammetric determination of 2,4-DCP.

**Table 1:** Comparison of MIL-101 (Fe)-NH_2_/GO/SPCE sensor with other sensors for electrochemical determination of 2,4-DCP.

Electrochemical sensor	Electrochemical method	Linear range	LOD	Reference
Cross-linked polyaniline/GO-oxidized single-walled carbon nanotubes/glassy carbon electrode (GCE)	DPV	0.05–1.2 μM	4.2 nM	[3]
Cu-based MOF/electrochemically reduced GO/GCE	DPV	1.5–24 μM	0.083 μM	[14]
Rutin-reduced GO/TiO_2_ nanocomposite/GCE	DPV	5–150 μM	0.02 μM	[17]
Au nanoflakes/ZrO_2_/GCE	DPV	1.5–24 μM	0.056 μM	[74]
*β*-cyclodextrin functionalized ionic liquid/carbon paste electrode (CPE)	Amperometry	4–100 μM	1.2 μM	[75]
Tyrosinase/MWCNTs/poly (diallyldimethylammonium chloride) (PDDA)/GCE	Amperometry	2–100 μM	0.66 μM	[76]
MoS_2_-ionic liquid-Au-Ag nanorods/GCE	DPV	0.01–50 μM	0.0026 μM	[77]
MIL-101 (Fe)-NH_2_/GO/SPCE	DPV	0.001–440.0 μM	0.5 nM	This work

### 3.6 Application of MIL-101 (Fe)-NH_2_/GO/SPCE in analysis of 2,4-DCP in the water samples

To validate the developed sensor in practical analysis, determination of 2,4-DCP was performed in the tap water and well water samples. Firstly, the water samples were analyzed to determine the presence of 2,4-DCP, but it was found that the target compound (2,4-DCP) was not detected in the water samples. Then, the water samples were spiked with specific concentrations of 2,4-DCP and analyzed using the standard addition method by DPV. From the obtained results in Table 2, the good recoveries for the spiked concentrations of 2,4-DCP (97.5–104.4 %), indicated the high accuracy of this sensor for 2,4-DCP determination. Furthermore, the calculated relative standard deviations (RSDs) from three measurements of each concentration of 2,4-DCP (n = 3) was less than 3.4 %, which reveal the high precision of the designed sensor in the present study.

**Table 2:** Determination of 2,4-DCP in the water samples at MIL-101 (Fe)-NH_2_/GO/SPCE (n = 3).

Sample	Spiked Concentration of 2,4-DCP (μM)	Found Concentration of 2,4-DCP	Recovery (%)	RSD (%)
**Tap water**	0	–	–	–
	4.0	3.9	97.5	3.4
	6.0	6.2	103.3	1.9
	8.0	8.1	101.2	2.9
	10.0	9.8	98.0	2.2
**Well water**	0	–	–	–
	5.0	5.1	102.0	1.8
	7.0	6.9	98.6	3.1
	9.0	9.4	104.4	2.4
	11.0	10.9	99.1	2.7

## 4 Conclusion

In summary, this study described the development of an electrochemical sensor using SPCE modified with MIL-101 (Fe)-NH_2_/GO nanocomposite for voltammetric determination of 2,4-DCP. The MIL-101 (Fe)-NH_2_/GO nanocomposite was successfully prepared and characterized by different techniques (FE-SEM, XRD, and EDX). The CV studies in PBS (0.1 M − neutral pH) demonstrated that MIL-101 (Fe)-NH_2_/GO/SPCE sensor was highly active for oxidation reaction of 2,4-DCP. A higher current response was observed for 2,4-DCP after modifying the SPCE with the MIL-101 (Fe)-NH_2_/GO nanocomposite along with a shift in the oxidation potential to less positive potentials. DPV measurements were conducted on MIL-101 (Fe)-NH_2_/GO/SPCE to perform quantitative measurements. Based on the results, the developed sensor demonstrated high sensitivity towards 2,4-DCP by presenting linearity range of 0.001–440.0 μM with a low LOD of 0.5 nM. At finally, using the standard addition method, the modified SPCE was successfully used for quantitative determination of 2,4-DCP in tap and well water samples. The MIL-101 (Fe)-NH_2_/GO nanocomposite modified SPCE is a promising electrochemical sensing platform to improve the electroanalysis of compounds in environmental monitoring.

## CRediT authorship contribution statement

**Hadi Beitollahi:** Writing – review & editing, Writing – original draft, Formal analysis. **Somayeh Tajik:** Writing – review & editing, Writing – original draft, Formal analysis. **Fariba Garkani Nejad:** Writing – review & editing, Writing – original draft, Formal analysis.

## Data availability statement

Data will be made available on request. For requesting data, please write to the corresponding author.

## Declaration of competing interest

The authors declare that they have no known competing financial interests or personal relationships that could have appeared to influence the work reported in this paper.
